# Cannabidiol Does Not Impact Acute Anabolic or Inflammatory Signaling in Skeletal Muscle *In Vitro*

**DOI:** 10.1089/can.2021.0132

**Published:** 2022-10-12

**Authors:** Henning T. Langer, Alec Avey, Keith Baar

**Affiliations:** ^1^ Department of Physiology and Membrane Biology, Physiology and Behavior, University of California, Davis, California, USA.; ^2^ Department of Neurobiology, Physiology and Behavior, University of California, Davis, California, USA.; ^3^ VA Northern California Health Care System, Mather, California, USA.

**Keywords:** muscle, CBD, mTORC1, NF-κB, inflammation, C2C12

## Abstract

**Background:**

Cannabidiol (CBD) is becoming increasingly popular for the treatment of clinical conditions including as an aid for muscle recovery. Previous work demonstrated that CBD exhibited mild effects on skeletal muscle, with a tendency to increase anabolic signaling and decrease inflammatory signaling.

**Methods:**

To gain mechanistic insight and extend these findings, we conducted a set of experiments using C2C12 myotubes.

**Results:**

Increasing the dose of CBD (1–5 μM) provided with insulin-like growth factor 1 (IGF-1) showed no effect on anabolic signaling through mTORC1 (S6K1 [Thr389], *p*=0.27; rpS6 [Ser240/244], *p*=0.81; or 4E-BP1 [Thr37/46], *p*=0.87). Similarly, inflammatory signaling through nuclear factor kappa B (NF-κB) (p105, *p*=0.88; p50, *p*=0.93; or phosphorylated p65 [Ser536], *p*=0.84) in response to tumor necrosis factor α (TNFα) was unaffected by CBD (2.5 μM), whereas dioscin, a natural product that blocks NF-κB signaling, reduced p105 and phosphorylated p65 (Ser536) compared with the TNFα and the TNFα + CBD condition (*p*<0.01 and *p*<0.05, respectively). Finally, cannabinoid receptor type 1 (CB1) receptor levels were measured in C2C12 cells, murine skeletal muscle, cortex, and hippocampus. Although CB1 was not detectable in muscle cells or muscle tissue, high levels were observed in brain tissue.

**Conclusion:**

In conclusion, CBD does not directly modulate anabolic or inflammatory signaling in myotubes *in vitro*, which can likely be explained by the lack of functional receptors.

## Introduction

Cannabidiol (CBD) is the main chemical compound in the *Cannabis sativa* plant. It has been shown to have several medical properties such as being an anticonvulsant, analgesic, and anti-inflammatory.^1,2^ CBD was recently approved by the Food and Drug Administration (FDA) for the treatment of epilepsy and symptoms of multiple sclerosis. Other reports have shown that CBD has promise for the treatment of neuromuscular disorders.^3^ Beyond its clinical application, the increasing national and international legalization of CBD has caused a rise in medicinal use for the treatment of pain, sleeplessness, or as a recovery aid after exercise.^4^

Recently, we investigated the effect of acute administration of CBD on molecular signaling in skeletal muscle after eccentric exercise in rats.^5^ We found that despite a relatively high dose of intraperitoneal CBD, the overall effect on protein levels in skeletal muscle after exercise was modest. There was a tendency for increased anabolic signaling through mTORC1 and a concomitant decrease in inflammatory signaling through nuclear factor kappa B (NF-κB) with the administration of CBD. Interestingly, previous research into drugs such as ibuprofen has indicated that blunting inflammatory signaling could have adverse effects on skeletal muscle adaptations and muscle growth in response to acute and chronic exercise.^6,7^

 Therefore, finding a treatment option that decreases inflammation and pain without hampering anabolic signaling, muscle remodeling, growth, or other adaptations would be of great interest to a wide range of individuals. However, robust evidence for such an effect of CBD on skeletal muscle is lacking and mechanistic insights are sparse. For example, it is currently unknown whether the decrease in inflammatory markers that we previously observed in muscle *in vivo* was the result of a direct effect of CBD on muscle or an indirect effect on inflammatory cells.

To determine whether the decreased inflammation and the tendency to increase anabolism *in vivo* were the results of a direct action on muscle cells, we conducted a set of cell culture experiments in C2C12 myotubes. We hypothesized that CBD would amplify anabolic signaling through the mTORC1 axis and decrease inflammatory signaling through NF-κB.

## Materials and Methods

### Cell culture

C2C12 myoblasts were cultured in growth media (high glucose Dulbecco's modified Eagle medium [DMEM], 10% fetal bovine serum, 0.1% penicillin) until they reached 95–100% confluence at which time, they were shifted into differentiation media (DM; high glucose DMEM, 2% horse serum, 0.1% penicillin) to promote the differentiation and fusion of cells to form myotubes. All experiments were conducted on fully formed myotubes 5 days after the introduction of DM.

### Anabolic signaling

**FIG. 1.**
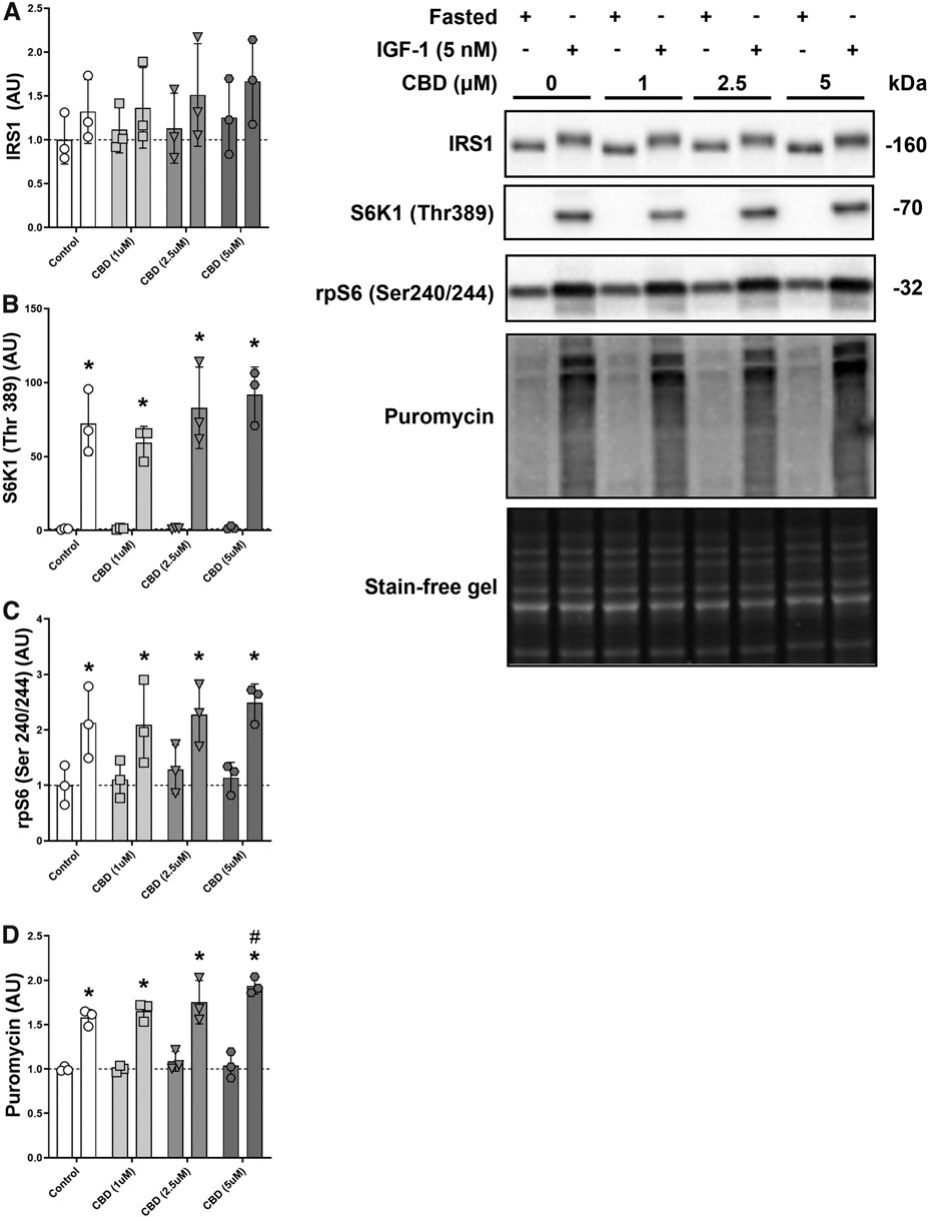
CBD and anabolic signaling in a fasted state and after IGF-1 treatment. Protein levels of IRS1 **(A)**, phosphorylated S6K1 (Thr389) **(B)**, phosphorylated rpS6 (Ser240/244) **(C)**, and puromycin **(D)** with increasing dosages of CBD (1–5 μM) in C2C12 myotubes in a fasted condition on PBS +2% horse serum or after 30 min on DM + IGF-1 (5 nM). *p-Value <0.05 compared with the fasted control, ^#^*p*-value <0.05 compared with the IGF-1 control condition. *n*=3 biological replicates. CBD, cannabidiol; DM, differentiation media.

The first dose–response relationship experiment for CBD and anabolic signaling was assessed using a previously published protocol with modifications (Fig. 1).^8^ Myotubes plated in six-well plates were fasted for 15 min by replacing DM with starvation media (SM; phosphate-buffered saline [PBS], 2% horse serum, and increasing dosages of CBD [0, 1, 2.5 and 5 μM]). The CBD product (Amber Metric, Santa Rosa, CA) was laboratory tested and hemp derived (99.8% purity). After the 15 min fasting period, SM in half of the wells was replaced with DM containing insulin-like growth factor 1 (IGF-1) (5 nM; PeproTech, NJ) and increasing dosages of CBD for a duration of 30 min before the cells were collected.

**FIG. 2.**
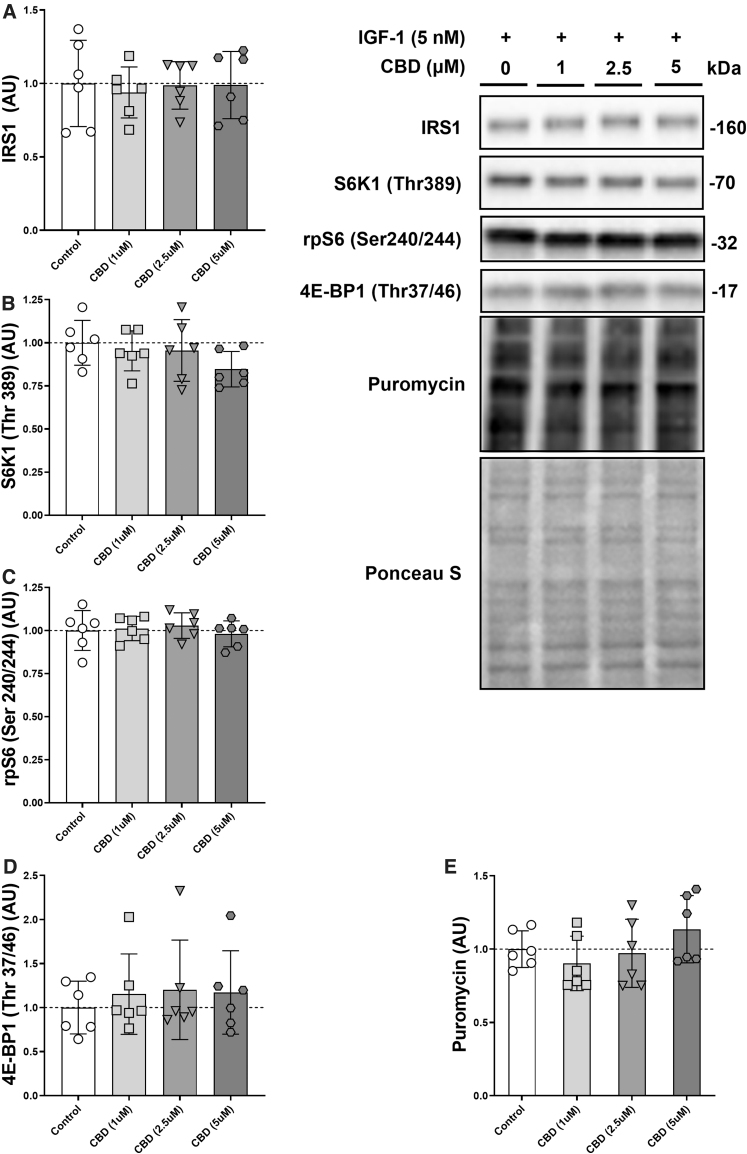
Dose–response relationship between CBD and mTORC1 signaling. Protein levels of IRS1 **(A)**, phosphorylated S6K1 (Thr389) **(B)**, phosphorylated rpS6 (Ser240/244) **(C)**, phosphorylated 4E-BP1 (Thr37/46) **(D)**, and puromycin **(E)** with increasing dosages of CBD (1–5 μM) in C2C12 myotubes after 60 min on DM + IGF-1 (5 nM). *n*=6 biological replicates.

To improve statistical power and based on the absence of baseline differences in the first experiment, we grew the cells on 24-well plates and omitted the starved condition from the second dose–response experiment (Fig. 2). Instead, DM in all wells was replaced with DM + IGF-1 (5 nM) with increasing dosages of CBD (0, 1, 2.5, and 5 μM) for 60 min before collection.

### Natural product screen for inhibitors of the NF-κB activity

To identify natural products that could decrease the activity of NF-κB in response to tumor necrosis factor α (TNFα), NF-κB reporter (luc)-HEK293 cells (BPS Bioscience, San Diego, CA) containing the firefly luciferase gene driven by four copies of NF-κB response element were treated with 10 ng/mL TNFα. Following treatment for 6 h, cells were collected in passive lysis buffer and luciferase activity was determined using a GloMax(R) 20/20 Luminometer System w/dual injectors (Promega, Madison, WI). To find NF-κB inhibitors, 146 structurally diverse, bioactive, and cell permeable natural products (Selleck Chemicals, Houston, TX) were added together with TNFα at a final concentration of 10 μM. Of the six natural products that decreased the NF-κB activity by more than 85%, the effect of increasing doses was determined. Data are presented for one of these natural products (dioscin).

### Inflammatory signaling

**FIG. 3.**
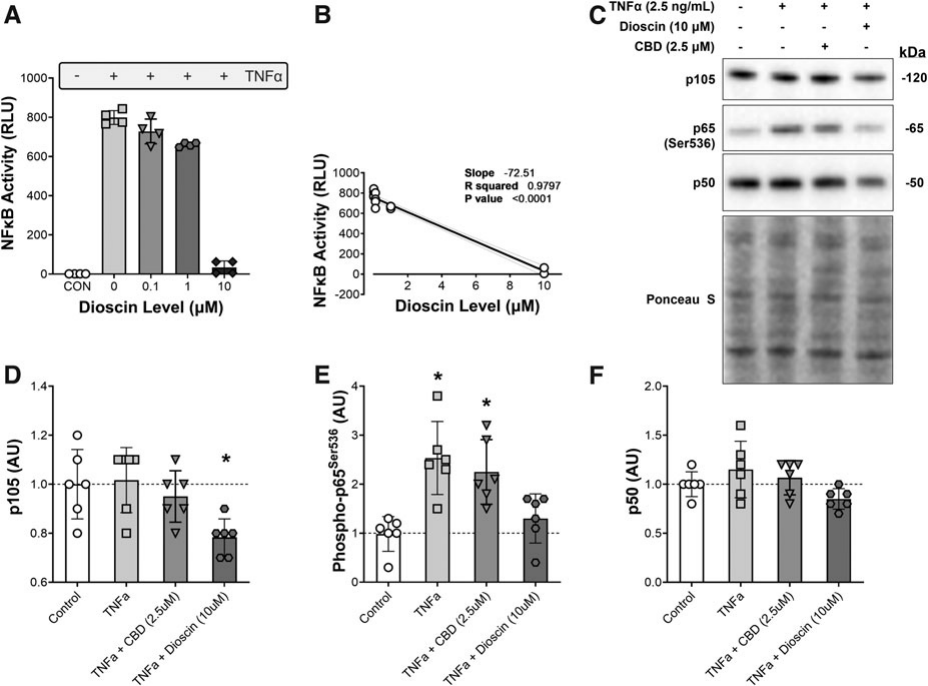
CBD does not decrease inflammatory signaling through NF-κB after TNFα treatment. **(A)** The transcription activity of NF-κB in control HEK293 cells and following treatment with TNFα and increasing doses of the steroidal saponin dioscin. **(B)** The dose–response relationship between dioscin and NF-κB activity. **(C)** Representative pictures of western blot analysis. Levels of **(D)** NF-κB p105, **(E)** phosphorylated NF-κB p65 (Ser536), and **(F)** and NF-κB p50 in C2C12 myotubes after 60 min in DM, or following treatment with TNFα (2.5 ng/mL), TNFα + CBD (2.5 μM), or TNFα + dioscin (10 μM). *p-Value <0.05 compared with the control and the TNFα condition **(A)**, and *p*-value <0.05 compared with the control and the TNFα + dioscin condition **(B)**. *n*=6 biological replicates. NF-κB, nuclear factor kappa B; TNFα, tumor necrosis factor α.

For the experiment on CBD and acute inflammatory signaling, C2C12 cells were differentiated into myotubes in 24-well plates. On the day of the experiment, existing media in the wells was replaced with fresh DM that contained no additional compounds, DM + TNFα (2.5 ng/mL; PeproTech), DM + TNFα + CBD (2.5 μM), or DM + TNFα + the phytochemical dioscin (10 μM; Selleck Chemicals) as a positive control. Cells were treated for 1 h before collection (Fig. 3).

### Cannabinoid receptor type 1 protein levels

**FIG. 4.**
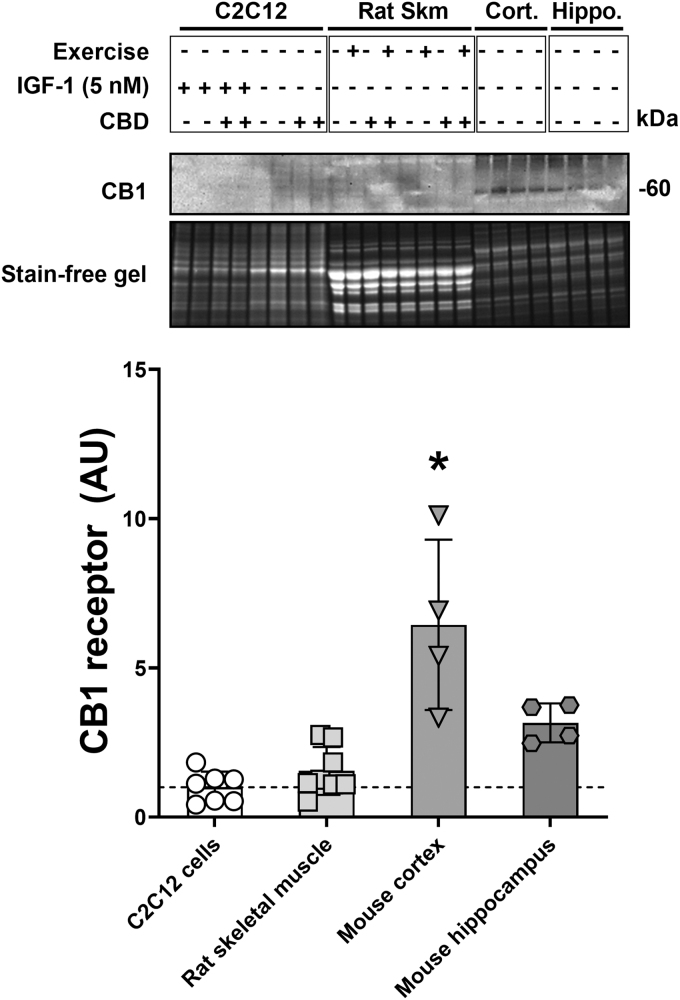
CB1 receptor protein levels in C2C12 cells, rat skeletal muscle, and murine brain tissue. Protein levels of CB1 in C2C12 cells with and without IGF-1 treatment (5 nM) in the presence or absence of CBD (5 μM), rat tibialis anterior after eccentric exercise in the presence or absence of CBD (100 mg/kg body weight administered intraperitoneally), mouse cortex, and mouse hippocampus. *p-Value <0.05 compared with all other conditions. *n*=8 biological replicates (C2C12 cells), *n*=8 animals (rat tibialis anterior), *n*=4 animals (murine cortex), and *n*=4 animals (murine hippocampus). CB1, cannabinoid receptor type 1.

To compare protein levels of cannabinoid receptor type 1 (CB1) between skeletal muscle *in vitro* and *in vivo* with a positive control (brain tissue), we used cell culture samples from the experiments above and analyzed them alongside of rat tibialis anterior tissue from previous CBD experiments^5^ and murine cortex and hippocampus samples (Fig. 4).

### Cell collection and Western blot analysis

For assessment of global muscle protein synthesis via the SUnSET method, 50 μL of puromycin (45 μM; Research Products International, IL) was added to every cell culture well 5 min before collection. Upon collection, all wells were placed on ice and washed three times with ice-cold PBS. For six-well plates, 100 μL of ice-cold sucrose lysis buffer was added to each well after the last wash, as described previously.^8^ Cells were then scraped off the plate using a spatula and collected in an Eppendorf tube. After centrifuging them at 10,000 *g* for 5 min at 4°C, 80 μL of the supernatant was transferred to a fresh tube and 20 μL of 4×Laemmli sample buffer (LSB) was added. The samples were then vortexed, heated at 100°C for 5 min, and finally stored at −30°C until Western blot analysis was completed. For the 24-well plate experiments, the collection protocol was identical except for two modifications: after the last washing step with ice-cold PBS, 75 μL of 1×LSB was directly added to each well before the samples were transferred to a fresh tube. The samples were then briefly sonicated before boiling and storage at −30°C until further analysis.

Western blotting was carried out as described previously.^8^ Briefly, for the cell culture experiments, 10 μL of sample was loaded per lane, run for 40 min at 200 V, and transferred onto polyvinylidene fluoride or nitrocellulose membrane in an ice-cold transfer buffer at 100 V for 30 min. For the rodent skeletal muscle and brain samples, 10 μg of protein per lane was loaded. Membranes were blocked in 1% fish skin gelatin dissolved in Tris-buffered saline with 0.1% Tween-20 for 30 min and probed with the primary antibody overnight. The following antibodies were used: Cell Signaling (Cell Signaling Technology, Danvers, MA): S6K1 (Thr389) (No. 9205; lot 16), ribosomal protein S6 (Ser240/244) (No. 5364), 4E-BP1 (Thr37/46) (No. 2855), NF-kB p65 (Ser536) (No. 3033; lot14), NF-kB p105/p50 (No. 13586), CB1 receptor (No. 93815); Millipore Sigma (Merck Group): IRS1 (No. 06-248; lot 2465193), puromycin (No. MABE343). Levels of each protein were normalized to total protein content per lane as assessed via Ponceau S staining of the membrane or a fluorescent gel.^9^

### Statistics

Data analysis was carried out using GraphPad Prism 9 (GraphPad Software, San Diego, CA). One- or two-way analysis of variance combined with Dunnett's (one-way analysis of variance [ANOVA]; Fig. 2) or Tukey's (one-way ANOVA and two-way ANOVA; Figs. 1, 3, and 4) multiple comparison test to allocate differences *post hoc* were applied depending on the number of groups and variables. The alpha level was set at *p*=0.05; *p*-values <0.05 were deemed statistically significant, and *p*-values between 0.05 and 0.1 are described as trends.

## Results

### CBD and anabolic signaling in a fasted state and after IGF-1 treatment

IRS1 protein showed a distinct upwards shift in the gel indicating post-translational modification. The absolute amount of protein tended to increase with IGF-1 treatment (*p*=0.06) and CBD did not affect this process (CBD: *p*=0.65 and interaction: *p*=0.99; Fig. 1A). Phosphorylated S6K1 (Thr389) levels increased significantly with IGF-1 treatment (*p*<0.0001) without significant effect of CBD (*p*=0.27) or an interaction effect (*p*=0.3). No differences at baseline or after IGF-1 treatment between the different CBD conditions could be detected via *post hoc* testing (Fig. 1B). Similarly, phosphorylated rpS6 (Ser240/244) increased with IGF-1 treatment (*p*<0.0001) without an effect of CBD (*p*=0.76) or an interaction effect (*p*=0.91; Fig. 1C). Finally, global protein synthesis as assessed via puromycin increased significantly with IGF-1 treatment (*p*<0.0001) and showed a trend for an effect of CBD (*p*=0.06) but no interaction effect (*p*=0.18). *Post hoc* testing determined a difference between the IGF-1 control condition and the IGF-1 CBD (5 μM) condition (*p*<0.05; Fig. 1D).

### Dose–response relationship between CBD and mTORC1 signaling

To increase technical replicates in each trial, the starved conditions were omitted and the effect of CBD on the IGF-1 response was determined. Total IRS1 protein levels did not differ significantly (Fig. 2A). Similarly, S6K1 (Thr389), rpS6 (Ser240/244), 4E-BP1 (Thr37/46), and puromycin failed to show a main effect of CBD (*p*=0.27, *p*=0.81, *p*=0.87, and *p*=0.26, respectively) or *post hoc* group differences (Fig. 2B–E).

### CBD does not decrease inflammatory signaling through NF-κB after TNFα treatment

To investigate NF-κB signaling, natural product inhibitors of NF-κB transcription were initially identified in HEK293 NF-κB reporter cells. Of 146 structurally diverse natural products, 6 decreased the NF-κB activity >85%. Of these factors, we focused of dioscin, a natural steroidal saponin that inhibited NF-κB activation by TNFα in a dose-dependent manner (Fig. 3A, B). C2C12 cells were then treated with 2.5 ng/mL TNFα in the presence or absence of 2.5 μM CBD or 10 μM dioscin (Fig. 3). We found a main effect for p105 protein levels (*p*<0.01) with group differences between the control condition and cells that were treated with TNFα and dioscin (*p*<0.05) as well as a difference between the TNFα condition and the TNFα plus dioscin condition (*p*<0.05; Fig. 3D), whereas CBD had no effect. Phosphorylated p65 (Ser536) also showed a main effect (*p*<0.001) with various group differences. TNFα treatment increased phosphorylated-p65 compared with the control condition (*p*<0.001). Similarly, TNFα in conjunction with CBD increased phosphorylated-p65 levels significantly (*p*<0.01), whereas phosphorylated-p65 did not increase with TNFα and dioscin treatment. Accordingly, phosphorylated-p65 levels with TNFα or TNFα plus CBD were significantly higher than with TNFα and dioscin (*p*<0.01 and *p*<0.05, respectively; Fig. 3E). There was no significant effect of any treatment on p50 levels, but a trend toward a main effect (*p*=0.07) and a trend toward a group difference between TNFα and TNFα plus dioscin (*p*=0.05; Fig. 3F).

### CB1 receptor protein levels in C2C12 cells, rat skeletal muscle, and murine brain tissue

Since CBD had modest effects on skeletal muscle *in vitro* and *in vivo*, levels of the main cannabinoid receptor (CB1) were determined in C2C12 cells and rat tibialis anterior muscle, each with and without CBD treatment. As a positive control, murine brain tissue from either the cortex or hippocampus where CB1 receptor is abundant was run in parallel. The predicted molecular weight of CB1 is 52.8 kDa, but the protein tends to run at around 60 kDa. CB1 was highest in the murine cortex, significantly less CB1 was observed in the hippocampus (*p*<0.01), and the protein was not detected in either the C2C12 myotubes or the rat TA muscle (Fig. 4).

## Discussion

In this study, we investigated the effect of CBD on anabolic and inflammatory signaling in C2C12 myotubes. We looked at the effect of CBD on mTORC1 signaling under fasted conditions and after addition of amino acids and IGF-1. We found that a range of 1–5 μM of CBD had no effect on anabolic signaling through the mTORC1-axis. However, we did detect a small increase in global protein synthesis via puromycin after IGF-1 treatment for the highest CBD dose. Since there were no baseline differences, the effect of CBD (5 μM) on puromycin after IGF-1 was small, and the technical replicates were limited (*n*=3; Fig. 2), we decided to repeat the experiment, double the technical replicates, and omit the fasted condition. In our follow-up experiment with increased statistical power (Fig. 3), the observed tendency of higher protein levels of mTORC1 signaling and global protein synthesis with increasing dosages of CBD disappeared.

To investigate the anti-inflammatory properties of CBD in muscle cell culture, we tested NF-κB signaling in the presence or absence of TNFα, together with CBD or a natural product inhibitor of NF-κB activation dioscin. Dioscin was identified as a potent inhibitor of TNFα using an NF-κB reporter cell line to screen 146 natural products. Compared with dioscin, CBD had little to no effect on inflammatory signaling through the NF-κB axis in muscle cells. Although dioscin decreased the phosphorylation and activation of p65 (Ser536), p105, and tended to decrease NF-κB p50, CBD had no effect on NF-κB signaling.

To determine whether the inability of CBD to affect anabolism or inflammatory signaling in C2C12 myotubes was the result of low receptor number, the primary CBD receptor CB1 was determined in C2C12 cells and skeletal muscle of rodents and compared with the cortex and hippocampus. Neither myotubes nor muscle demonstrated CB1 protein, and this likely explains the observation that CBD did not affect myotubes in cell culture and had modest effects on muscle *in vivo*. Previous studies on CB1 in muscle were able to detect CB1 in skeletal muscle cell culture and in rodent muscle samples.^10–12^ However, these studies used immunoprecipitation of specific subfractions to enrich CB1 in an effort to detect CB1 protein levels. It is possible that using these methods we would be able to detect CB1 in our samples. However, the current work clearly demonstrates that the cellular abundance of CB1 is orders of magnitude lower in muscle cells than in brain tissue. This observation is in line with earlier findings on the subject.^13,14^

A significant issue with any cell culture experiment is dosing and the translatability of findings to the *in vivo* setting. In our previous experiments in rats, we combined eccentric exercise with the injection of 100 mg CBD per kg bodyweight (intraperitoneal).^5^ Deiana et al. have measured plasma CBD levels after intraperitoneal injection of 120 mg per kg bodyweight in mice.^15^ Plasma levels peaked at 14 μg/mL at 120 min after injection, equivalent to about 45 mM. Since the dosage we used in our cell culture experiments is considerably lower (1–5 μM), it is possible that CBD would have started to exhibit effects at higher concentrations.

However, in a recent systematic review on CBD in cell culture, the authors concluded that CBD starts to negatively impact cell viability at doses higher than 2 μM and induces apoptosis at dosages higher than 10 μM.^16^ Therefore, we chose 5 μM as the upper limit of our dose–response studies. Indeed, we performed a subset of our experiments with 10 μM without seeing any beneficial effects compared with 5 μM. Similarly, going out to 24 h with the CBD treatment concomitantly with TNFα or before the addition of IGF-1 had no effect on NF-κB or mTORC1 signaling, respectively (data not shown).

Furthermore, it is important to remember that the effects of CBD on anabolic and inflammatory signaling in our *in vivo* experiments were small.^5^ This was the case despite a relatively high dose and an efficient route of administration, which could be associated with toxicity in chronic settings.^17–20^ Given this context, it appears unlikely that there is a direct physiological role for CBD in anabolic and inflammatory signaling within skeletal muscle tissue.

Even though our data indicate that direct effects of CBD on anabolic or inflammatory signaling in skeletal muscle *in vitro* are small, it is still possible that CBD could exert indirect effects *in vivo* through modulation of immune cells that interact with skeletal muscle or by causing systemic changes to circulating cytokines and hormones that affect the central nervous system, immune system, and other endocrinologically active tissues. Future research needs to determine whether such indirect effects could be clinically relevant and whether they can be elicited without requiring toxic dosages of CBD. Additionally, future projects will need to investigate in how far the effects of CBD on skeletal muscle may differ from other cannabinoids, endocannabinoids and analogs.

## Abbreviations Used

ANOVA: analysis of variance
CB1: cannabinoid receptor type 1
CBD: cannabidiol
CHRC: Cannabis and Hemp Research Center
DM: differentiation media
FDA: Food and Drug Administration
LSB: Laemmli sample buffer
NF-κB: nuclear factor kappa B
PBS: phosphate-buffered saline
RICH: Research Investments in Cannabis and Hemp
SLB: sucrose lysis buffer
SM: starvation media
TNFα: tumor necrosis factor α
